# Cross-Sectional Study Assessing HIV Related Knowledge, Attitudes and Behavior in Namibian Public Sector Employees in Capital and Regional Settings

**DOI:** 10.1371/journal.pone.0075593

**Published:** 2013-09-20

**Authors:** Til R. Kiderlen, Michael Conteh, Stephanie Roll, Stefanie Seeling, Stefan Weinmann

**Affiliations:** 1 Department of Medicine, Hematology/Oncology/Infectious Diseases/Rheumatology, Goethe University, Frankfurt, Germany; 2 Institute of Tropical Medicine and International Health, Charité–Universitätsmedizin Berlin, Berlin, Germany; 3 Multidisciplinary Research Centre, University of Namibia, Windhoek, Namibia; 4 Namibia Institute of Public Administration and Management, Windhoek, Namibia; 5 Institute for Social Medicine, Epidemiology and Health Economics, Charité–Universitätsmedizin Berlin, Berlin, Germany; 6 Berlin School of Public Health, Carité-Universitätsmedizin, Berlin, Berlin, Germany; 7 Deutsche Gesellschaft für Internationale Zusammenarbeit, Berlin, Germany; University of Buea, Cameroon

## Abstract

The study objective was to assess the current status of HIV knowledge, attitudes and behavior (KAB) among employees of Namibian ministries. As most HIV campaigning takes place in the capital of Windhoek, an additional aim was to compare Windhoek to four regions (Hardap, Erongo, Oshana, and Caprivi). Between January and March 2011 a cross-sectional survey was conducted in two Namibian ministries, with participants selected randomly from the workforce. Data collection was based on questionnaires. 832 participants were included in the study (51.6% male). Nearly 90% of participants reported to have been tested for HIV before. Knowledge about HIV transmission ranged from 67% to 95% of correct answers, with few differences between the capital and regions. However, a knowledge gap regarding HIV transmission and prevention was seen. In particular, we found significantly lower knowledge regarding transmission from mother-to-child during pregnancy and higher rate of belief in a supernatural role in HIV transmission. In addition, despite many years of HIV prevention activities, a substantial proportion of employees had well-known HIV risk factors including multiple concurrent partnership rates (21%), intergenerational sex (19%), and lower testing rates for men (82% compared to women with 91%).

## Introduction

After the first reported case in Namibia in 1986, HIV rapidly became a generalized epidemic in the country, with prevalence rising from about 4% in 1992 to about 19% in 2010 [1]. Accordingly, HIV has been estimated to account for over 50% [2] of all deaths in Namibia in 2002. More recently, this number has dropped since then to 23% [3]. This is most likely due to the high coverage of antiretroviral therapy with about 90% of people in need being treated [4], nevertheless, the disease is still one of the major causes of illness and death in the country. Although HIV prevalence rates in Namibia are known to differ between the capital and regional settings, substantial variation between single regions, from 4 to 36%, also occur [1]. Such differences are likely to influence or to be influenced by HIV related knowledge, attitude, and behavior. In order to design and implement a successful HIV prevention program, it is therefore essential to attain a proper understanding of regional differences in perception and behavioral patterns. Despite years of HIV campaigning in Namibia however, such data is limited.

The public sector is of special interest for HIV prevention as it is the largest employer in Namibia with approximately 85.000 employees [5]. Their scope of influence is even more substantial when one considers that public sectors prevention activities include utilizing social networks and largely affects the relatives of its employees as well. Furthermore, the public sector is a good cross-sectional representation of the entire country as it is composed of numerous different social groups and income categories, from the unskilled worker to the upper management.

There is no valid data on HIV prevalence in the public sector. However, a study of the Office of the Prime Minister (OPM) extrapolated available data from other sources to the public sector employees to assess the impact of HIV and AIDS. The study estimated a prevalence of about 15% [6]. It is therefore reasonable that one component of the “National Strategic Framework for HIV/AIDS response in Namibia” (NSF) is to establish HIV Workplace Programs (WPP) [3] in the public sector. The programs commonly focus on HIV prevention and the provision of, and referral to, HIV testing, care and support services.

The present study was conducted in the public sector in Namibia in the context of the implementation of WPPs. It aimed at providing information on HIV related knowledge, attitudes, and behavior of ministerial employees. The study’s objective was to identify gaps and needs with regard to HIV, and to provide baseline data for future monitoring and evaluation of workplace programs. In addition, as most HIV campaigning takes place in the capital, we aimed to compare the capital Windhoek and four rural regions (Hardap, Erongo, Oshana, and Caprivi).

## Methods

This cross-sectional study was part of a larger survey including the public and private sector in Namibia. The present manuscript reports the public sector results only. The study was conducted between January and March 2011. The study assessed the current status of knowledge, attitudes and behavior (KAB) regarding HIV and AIDS among public employees. Results are compared between the capital (Windhoek) and four different rural regions (Hardap, Erongo, Oshana, and Caprivi).

### Ethics statement

The Namibian national ethical committee for health research in the Ministry of Health and Social Services granted their ethical approval prior to conducting this study. Questionnaire completion was anonymous and participants provided informed consent by marking a box at the first page of the questionnaire after introduction on the purpose of the study and voluntariness of participation was given. This consent procedure was approved by the ethical committee.

### Data collection and questionnaire

We used a knowledge, attitudes and behavior (KAB) survey questionnaire that was designed in close cooperation with regional partners (UNDP, UNAIDS, Pharmaccess, Namibian Business Coalition on AIDS (NABCOA), Ministry of Health and Social Services (MoHSS), OPM, University of Namibia (UNAM), and the Deutsche Gesellschaft für Internationale Zusammenarbeit (GIZ)). These agencies built a Technical Working Group (TWG) which monitored the study process. The questionnaire design met international standards and recommendations [7–9] and took into account previously used and established questionnaires in Namibia [10–12]. The questionnaire was designed in English and included multiple-choice as well as few open-ended questions. The questionnaire was then translated into the local languages Afrikaans, Oshiwambo, Otjiherero and Silozi. Independent persons did two-way translations from English into the local language and back into English, in order to assure appropriateness of the translation. Items which were potentially influenced by language were discussed with the fieldworkers, leading to minor changes of the translation. The questionnaire was composed of 117 questions which were organized into 6 subject areas. Literate participants filled out the questionnaire on their own (paper pencil version). Illiterate participants answered the same questions through an interview split in two parts. First, non-sensitive questions were completed with support of the field worker. For the second part, which contained sensitive questions, an indirect interview was conducted with the fieldworker reading and the participant answering them using symbols behind a blind screen, thus maintaining anonymity. To reduce interviewer bias, fieldworkers were extensively trained for data collection. This approach was pre-tested and was considered useful and appropriate. After completion, participants deposited their anonymous questionnaires in a locked and sealed ballot box.

### Selection of workplaces and participants

The two selected ministries are partners of German Development Cooperation. Both ministries have departments in every region of Namibia and have implemented a WPP. Regions were selected in order to represent the different settings of the country, covering high HIV prevalence regions (Oshana and Caprivi; HIV prevalence between 25 and 36%), middle (Erongo; 15-20%), and low HIV prevalence regions (Hardap; 11-14%) [1]. Regional distribution and the interests of the research partners were also considered in the selection process.

Participants were selected using a random sample based on employment lists. All employees of the participating ministries were eligible for this study, regardless of age, sex, education, or language spoken

### Study procedure

A pilot study was conducted in advance, leading to minor changes in study and questionnaire design. For the main study, field workers were trained on study methodology and received a comprehensive introduction to the study guidelines on data collection and confidentiality measures. Four field workers were trained for the first study exercise in the capital and were supervised by the principal researcher. Regional teams were organized according to their language background. These teams consisted of three experienced field workers and a supervisor. At the beginning of the data collection, selected employees were comprehensively educated about the aim and methodology of the study and about confidentiality issues in order to enable informed consent and minimize possible misconceptions. If a selected person was absent, she or he was replaced by another employee only if the provided employees list was outdated and the participant no longer worked for the ministry (i.e. left the company, retirement, death, etc.) or was transferred to a region not included in the study. Potential replacements were restricted to persons filling the vacancy (first preference), or persons of the same department, the same job category and the same sex (second preference).

### Data processing and statistical analyses

The sample size calculation was based on the expected difference in the ‘percentage of participants ever being tested for HIV’ (primary outcome measure) between the two regions (capital or non-capital). The expected difference was based on the results of the pilot study HIV testing rates in the capital of 95% and in the regions of 89%. The chosen parameter “ever been tested for HIV” is a simple but useful indicator for HIV related behavior. Changing behavior towards safer sex is an important aim of HIV campaigning. To achieve this aim, many behavior change interventions try to increase knowledge and attitudes related to HIV. For the sample size estimation, we used a two-sided Chi-squared test for the primary outcome with 80% power, a level of statistical significance of 0.05 (two-sided), and a rate of dropout/withdrawal of 5%. The calculation was performed using nQuery Advisor 6.02. The sample size was estimated at 353 participants per group (capital and region). To account for difficulties in the recruitment, 600 participants for each group were sampled.

Data was double-entered by two independent trained persons using EpiData 3.1^©^ Data processing and analysis was with done with STATA^®^ 10.0.

Characteristics were compared using Fisher Exact Test. For group comparisons, the answer “Not sure” was treated as an incorrect answer, if not stated differently. Odds Ratios (OR) with 95% confidence intervals (CI) were calculated using logistic regression. Sociodemographic characteristics were controlled for using multivariable analysis. Percentages were rounded and minor rounding discrepancies may occur. The total numbers differ between the items due to missings or unidentifyable marks. All tests were two-sided, at a significance level of 0.05. We did not adjust for multipe testing.

## Results

### Sociodemographics

A total of 832 questionnaires were completed, 443 in Windhoek and 389 in the four regions (Hardap 89, Erongo 63, Oshana 121, Caprivi 116). Of the randomly selected participants, 324 (27%) could not participate due to reasons related to their job (workshop, duty travel, leave), 43 (3.6%) were no longer employed (transferred, retired, dead), 1 (0.08%) was on sick leave, and 4 (0.3%) rejected to participate. There was no evidence of differences in sociodemographic baseline data between randomly selected employees and those who actually participated. Among included participants, 17 (5.2%) were replacements. Participants’ characteristics are displayed in Table **1**. The female to male ratio was equally distributed with about 50% men and women. There were less male in the capital than in the regions (46 vs. 58%, *p*=0.001). The majority of the participants were in the age group between 30 and 39 years (30.6%) and 40-49 years (34.3%). Participants in the regions were on average older (50.1% with at least 40 years old in the capital and 58.9% in the regions, *p*=0.023). Nearly half of participants reported that they were married (48.3%), followed by single (34.3%), cohabitating (10.0%), widowed (4.1%) and divorced (3.4%). About 90% of participants were literate, with higher literacy rates in the capital than in the regions (92% vs. 87%, *p*=0.016).

**Table 1:** Demographic data of study participants, in total and separately for Windhoek and the regions (Hardap, Erongo, Oshana, Caprivi), given as frequencies (n) and percentage (%).

	**Total**	**Capital**	**Regions**	***p-value***
	**n (%**)	**n (%**)	**n (%**)	
**Ministry**				
Ministry 1	384 (46.2)	168 (37.9)	216 (55.5)	
Ministry 2	448 (53.9)	275 (62.1)	173 (44.5)	
**Total**	**832**	**443**	**389**	**0.000**
**Sex**				
Male	411 (51.6)	196 (46.0)	215 (58.1)	
Female	385 (48.4)	230 (54.0)	155 (41.9)	
**Total**	**786**	**426**	**370**	**0.029**
**Age (years**)				
Up to 19	1 (0.1)	0 (0.0)	1 (0.3)	
20-29	118 (14.7)	77 (17.9)	41 (10.9)	
30-39	246 (30.6)	134 (31.2)	112 (29.9)	
40-49	276 (34.3)	142 (33.0)	132 (35.7)	
50-59	160 (19.9)	74 (17.2)	86 (22.9)	
60 and over	4 (0.5)	3 (0.7)	1 (0.3)	
**Total**	**805**	**430**	**375**	**0.000**
**Marital Status**				
Married	388 (48.3)	198 (46.1)	190 (50.8)	
Divorced	27 (3.4)	17 (4.0)	10 (2.7)	
Widowed	33 (4.1)	13 (3.5)	18 (4.8)	
Cohabitating	80 (10.0)	46 (10.7)	34 (9.1)	
Single	276 (34.3)	154 (35.8)	122 (32.6)	
**Total**	**804**	**430**	**374**	**0.025**
**Literacy**				
Literate	746 (89.7)	408 (92.1)	338 (86.9)	
**Total**	**832**	**443**	**389**	**0.016**

### HIV risk perception

When asked “How high do you think is your risk of getting HIV?”, 219 of 710 participants (31%) who responded to this item perceived themselves at high risk, 122 (17%) at moderate, 233 (33%) at low, and 136 (19%) at no risk at all. In the regions significantly more people saw themselves at high risk (35% in the regions vs. 26% in Windhoek; *OR* 1.52, 95% CI 1.05-2.20, *p*=0.026). Risk perception also differed among regions. In the low HIV prevalence region of Hardap, significantly less self-declared HIV negative participants considered themselves at high or moderate risk compared to the capital (29% vs. 45%, *OR* 0.5, 95% CI 0.3-0.8, *p*=0.014). Risk perception did not differ significantly between Caprivi (39%, *p*=0.090) or Erongo (50%, *p*=0.086) and the capital. However, the odds of considering oneself at high or moderate risk were significantly higher in the high-prevalence region Oshana (81%) compared to Windhoek (*OR* 5.1, 95% CI 3.0-8.9, *p*<0.001). Sex (*OR* 1.02, 95% CI 0.8-1.8, *p*=0.873), marital status (*OR* 0.97, 95% CI 0.9-1.1, *p*=0.508), or age (*OR* 0.9, 95% CI 0.8-1.1, *p*=0.129) had no significant influence on risk perception. Participants assessing themselves at low or no risk were significantly more likely to not to have used a condom in their most recent sexual encounter (*OR* 2.5, 95% CI 1.8-3.4, *p*<0.001). This result remained stable when adjusted for marital status. Participants indicating to have had multiple concurrent sexual relations in the last 12 months were more likely to have assessed their risk of getting HIV to be high or moderate compared to participants without concurrent relationships (*OR* 1.9, 95% CI 1.3-2.8, *p*<0.001).

### Knowledge about HIV and AIDS

Most employees knew about the main routes of HIV transmission, with up to 95% correct answers (Table **2**). Nevertheless, in the regions only 76.8% correct answers regarding transmission by supernatural causes and only 62.7% correct answers regarding mother-to-child transmission during pregnancy were observed. This result was mainly influenced by the very low knowledge status in Caprivi where only 56% knew about mother-to-child transmission during pregnancy with participants being less likely to know about this transmission route compared to the capital (*OR* 0.5, 95% CI 0.4-0.8, *p*=0.005).

**Table 2:** Correct answers regarding HIV transmission, in total and separately for Windhoek and the regions (Hardap, Erongo, Oshana, Caprivi), given as percentage (%) and total number of answers per question (N).

	**Total**	**Capital**	**Regions**	***p-value***
	**% (N**)	**% (N**)	**% (N**)	
Transmission by sex	95.3 (802)	95.8 (430)	94.6 (372)	0.506
Transmission by shaking hands	93.3 (804)	93.0 (401)	93.6 (373)	0.780
Reduce risk by always using condoms	90.2 (805)	90.7 (431)	89.6 (374)	0.635
Healthy-looking person can have HIV	90.2 (813)	94.5 (435)	89.2 (378)	0.006
Risk reduction by sex with only one faithful, uninfected partner	88.2 (807)	88.5 (433)	88.0 (374)	0.828
Transmission by sharing food	87.8 (806)	88.6 (430)	87.0 (376)	0.517
Transmission by supernatural means	80.6 (805)	84.0 (430)	76.8 (375)	0.012
Mother-to-child transmission by breastfeeding	80.4 (801)	78.7 (428)	82.3 (373)	0.213
Transmission by mosquito bites	79.4 (801)	78.5 (427)	80.5 (374)	0.485
Mother-to-child transmission during pregnancy	66.7 (795)	70.2 (420)	62.7 (375)	0.029

About half of participants (49%) knew about risk reduction for HIV-infection after circumcision (Table **3**); regardless if male or female (51% vs. 48%, *p*=0.470), and place of residence (capital 48% vs. regions 52%, *p*=0.286). 114 out of 331 male participants (34%) stated to be circumcised. The rates differed widely among regions; ranging from 43% in the capital Windhoek, to about 30% in Hardap, Erongo and Oshana, and only 16% in Caprivi. Over one half (112 of 186; 60%) of uncircumcised men expressed a willingness to get circumcised.

**Table 3:** Circumcision in the public sector, in total and separately for Windhoek and the regions (Hardap, Erongo, Oshana, Caprivi), given as percentage (%) and total number of answers per question (N).

	**Public Sector**	**Capital**	**Regions**	***p-value***
	**% (N**)	**% (N**)	**% (N**)	
Circumcised (only men)	34.4 (331)	42.6 (162)	26.3 (169)	0.003
*No -* Willingness to get circumcised	60.2 (186)	58.8 (80)	61.3 (106)	0.763
Know circumcision reduces risk of HIV-infection	49.4 (793)	47.6 (424)	51.5 (369)	0.286

### HIV testing

98% of participants indicated they knew where to get tested for HIV, and 87% reported to have been tested before (Table **4**). Regarding the main outcome “ever been tested for HIV”, men reported to have been tested less often than women (82 vs. 91%, *p*<0.001). Among those tested, 61% indicated to have had a test less than 12 months ago; 20% had a test between 12 and 24 month ago, and 19% over 24 months ago. After testing, 97% of participants received the results of the last test. Furthermore, 81% of married or cohabitating participants said they knew the HIV-status of the partner. 81% of participants indicated they would uptake an HIV-test offered by the ministry itself, 6% were not sure and 13% would reject it.

**Table 4:** HIV-testing, in total and separately for Windhoek and the regions (Hardap, Erongo, Oshana, Caprivi), given as percentage (%) and total number of answers per question (N).

	**Total**	**Capital**	**Regions**	***p-value***
	**% (N**)	**% (N**)	**% (N**)	
Know where to get testing	97.5 (797)	97.0 (427)	98.1 (370)	0.367
*Ever be tested before*	86.5 (780)	89.2 (416)	83.5 (364)	0.027
*Yes* - Received the last test results	97.2 (666)	97.8 (365)	96.4 (301)	0.350
*Yes* - HIV positive	11.9 (603)	7.6 (331)	17.3 (272)	0.032
Know partners HIV-status	79.8 (396)	81.2 (207)	78.3 (189)	0.532
Would take a test offered by the ministry	80.8 (805)	80.1 (426)	81.5 (379)	0.654

### Sexual behavior

21% of participants indicated to have engaged in concurrent sexual relations in the last 12 months (Table **5**), without significant regional differences (19% capital, 23% regions, *p*=0.213). However, significantly more male than female (30% vs. 13%, *p*<0.001) and more participants below the age of 30 (27% vs. 17% for 30 years and more, *p*=0.002) reported concurrent sexual relationships. 48% of single men under 30 years old indicated to have engaged in concurrent sexual relationships. Singles were more likely to have engaged in concurrent partnerships compared to participants in stable relationships (married, cohabitating) (29% vs. 17%, *OR* 2.0, 95% CI 1.4-2.9, *p*<0.001). Still, 17% of married and cohabitating participants engaged in concurrent partnerships with 77% of them reporting condom use at the last sexual encounter. 19% of employees reported to have had inter-generational sex in the last 12 months (with a partner being 10 years older or younger). Results did not vary with gender (male 21%, female 18%; *p*=0.358). Further analyses showed that cohabitating (*OR 1.9; 95% CI 1.02-3.3; p=0.04*) and single employees (*OR 1.9; 95% CI 1.2-2.8; p*=0.002) were more likely to have had inter-generational sex than married, divorced or widowed persons; even if adjusted for age.

**Table 5:** Sexual behavior in the public sector, in total and separately for Windhoek and the regions (Hardap, Erongo, Oshana, Caprivi), given as percentage (%) and total number of answers per question (N).

	**Public Sector**	**Capital**	**Regions**	***p-value***
	**% (N**)	**% (N**)	**% (N**)	
Concurrent partnerships (last 12 months)	21.2 (760)	19.4 (412)	23.3 (348)	0.213
Inter-generational sex (last 12 months)	19.0 (788)	18.7 (418)	19.5 (370)	0.786

## Discussion

Although Namibia is a predominantly rural country, HIV prevention campaigning is centered in the capital city, Windhoek. This is likely largely due to logistical reasons, distance and availability of HIV prevention workers. This is true for the general population as well as for employees of ministries. Regional imbalances in HIV prevention generated some concerns with regard to possible differences in HIV related knowledge, attitudes and behavior between the employees in the capital and outside the capital and also with respect to the effectiveness of the campaigns. This was one reason why in the present study employees from the capital were calculated separately and compared to the regions. We found that differences in knowledge, attitudes and behavior between the capital and the four rural regions were small with the exception of Caprivi, which proved to be an outlier.

Knowledge regarding principal HIV transmission routes was found to be high in our study population. This corresponds to the findings of the 2006 Namibian Demographic Health Survey (DHS) and a study by the Nawa Life Trust reporting high levels of knowledge regarding HIV in Namibia [10,12]. An exception was knowledge of mother-to-child transmission of HIV. As the probability of transmitting the virus from mother to child ranges between 15% and 45% [13–15] without preventive interventions, this suggest a high need for education, especially in high prevalence regions. Furthermore, this study revealed an uncertainty and misconceptions in parts of the workforce regarding transmission routes such as transmission of HIV by sharing food, supernatural means and mosquito bites.

Male circumcision is a medical intervention which significantly lowers the risk of HIV transmission, as several studies have demonstrated [16]. According to the 2006 DHS, in Namibia about 20% of all men were circumcised, but rates do vary between 1% and 57% between regions [12]. In our male study population one third indicated to have been circumcised. As could be expected, circumcision rates varied largely between the regions, with all rates exceeding national survey numbers. Among the uncircumcised more than half expressed willingness to get circumcised and only about a third rejected this completely. This is encouraging for campaigns promoting circumcision; especially as willingness to circumcise was the same in high or low HIV prevalence regions.

With a reported average HIV prevalence of about 19% in Namibia [1], appropriate personal risk assessment and protective behavior is essential to stop the on-going spread of the disease. In this regards, a considerable share of 48% self-declared HIV-negative study participants indicated to be at high or moderate risk “of getting HIV”. According to the aim of the study design, the different study regions varied significantly with regard to location, cultural context and, predominantly, HIV-prevalence; results generally reflect these preconditions. Hence, risk perception differed significantly between the regions and was in general positively associated with regional HIV prevalence; with the exception of Caprivi, where risk perception was surprisingly low taking into account that Caprivi has the highest HIV prevalence in the country [1]. This finding is consistent with other misconceptions being more prevalent in Caprivi and may be a possible explanation for the high prevalence rates of this region. Other factors, such as Caprivi’s remote location, its status as a transit zone between four countries (Angola, Zambia, Botswana, and Zimbabwe), and weak infrastructure may also play a role.

Several studies have demonstrated that general infection risk depends on multiple personal factors including age, gender, and marital status [17,18]. In this study, these three variables did not affect HIV risk self-perception. Risk self-assessment needs to be translated into behavior change in order to be a marker of HIV risk, and is expected to be influenced by knowledge on transmission routes and employees’ own exposure. Many previous studies however have demonstrated a weak association between knowledge and behavior [19–21]. It is to note our findings suggest an association between HIV related knowledge and risk behavior. One of the major indicators of perceived risk resulting in behavior change is condom use. Accordingly, this study found participants with low or no perceived risk for HIV infection to be less likely to use condoms. This finding was independent of living in a stable relationship (married or cohabitating) or not. This highlights the need for comprehensive information about HIV transmission and prevention, and is an indicator of the potential impact of information campaigns enabling a realistic and reasonable risk assessment. In this regard, it is particularly interesting to investigate what influence the actual sexual behavior has on the risk perception of participants. We found that reported concurrent sexual relationships in the last 12 months were associated with a perceived high or moderate risk of getting HIV, which suggests a more realistic risk assessment in this population group. Accordingly, average condom use at the most recent sexual encounter was relatively high. This behavior may result from informed decision making: engaging in concurrent sexual relationships changed behavior of some participants.

An important factor for successful prevention and treatment of HIV is early and reliable HIV testing. In the participating ministries universal testing has been promoted for several years. 98% of the participants of our study indicated knowing where to get tested and 87% declared to have been tested for HIV before. Although these numbers are high, more than 10% had never been tested with lower rates in men compared to women (82% vs. 91%). This result is similar to DHS rates (87% vs. 92%) [12]. A high percentage of participants would accept an offer by the ministry itself to receive an HIV test. This indicates a trust in institutional confidentiality, and individuals not afraid of possible negative consequences with regard to their job. Given this result, offering voluntary counseling and testing (VCT) in ministerial institutions seems to be an important strategy as part of the WPP.

To investigate behavior patterns and perceptions regarding HIV transmission, participants assessed their sexual behavior. Concurrent sexual relations are seen as particularly problematic for the spread of the disease, as newly infected persons are highly infectious because of rapid virus replication in the first weeks [17]. Thus, infected persons may pass on the virus rapidly before knowing their own status. In our study, 21% of participants indicated concurrent sexual relations in the last 12 months. These numbers correspond to the results of the DHS (2000: 21%, 2006: 16%) [11,12], with higher rates in young, male and single employees. Even though 77% of employees with multiple concurrent partners reported condom use (2006 DHS: 66-74%), these numbers are high, taking in consideration the high HIV rates in Namibia. The potentially dramatic consequences of such behavioral risks should therefore be continually addressed and highlighted in future public health campaigns.

About 50% of participants reported having used a condom during the most recent sexual encounter. This rate is substantially higher than in the last 2006 DHS with only about 22% indicating to use condoms in general as a contraceptive method [12]. However, the DHS represents a nationwide household survey, covering all regions and settings; thus, representing a very diverse population. Additionally, data collection in the DHS was done with face-to-face interviews, potentially provoking underreporting in sensitive issues. A further exclusively urban household survey reported even higher condom use at the most recent sexual encounter (55-78%) [10]. This lends validity to our data, as our study includes only employees living mostly in an urban setting.

Limitations of this study include the methodological limits of a cross-sectional design, that can demonstrate associations, but cannot determine causality as well as the standard limitations of questionnaire and interview based surveys, including e.g. selection bias, reporting bias, translation bias. We tried to minimize potential bias by choosing a random sample of participants, anonymization, training of fieldworkers, etc. The questionnaire was quite detailed due to multiple questions the participating organizations and stakeholders wanted answered. Therefore, a potential reporting bias with less time and thoroughness regarding the last questions may have occurred. In our post-study validation we found no evidence for such an effect, however, observations during the study have compelled us to recommend shortened questionnaire for follow-up studies. Another potential reporting bias may be caused by the inclusion of illiterate participants. We tried to minimize barriers to answer sensitive questions using indirect interviews. Post-study validation did not find differences between literate and illiterate participants which could not be explained by socio-demographic factors. Nonetheless, the inclusion of illiterate participants remains challenging and requires careful preparation and training. Data for this study was collected in an environment where the workplace program (WPP) has already been implemented for years. Generalization of the results to employees of other Namibian ministries with no or different WPPs is therefore limited. The results of this study, however, might inform workplace programs in similar settings of high HIV prevalence. In an era of up scaling antiretroviral treatment with the aim to prevent new HIV infections, further research is necessary how to integrate this approach into workplace programs. Our results demonstrate the need to further investigate regional and cultural particularities, especially in Caprivi, to overcome barriers for prevention and treatment. The aspect of concurrent partnerships also needs to be better understood to appropriately adjust HIV prevention measures, as it is considered as one of the key factors for high HIV prevalence rates. Considering the limited prospects of rapid success by promotion of behavioural change, best-practice for promotion and implementation of more technical prevention strategies (condoms and VCT) have to be evaluated. This is especially true for the most at risk groups; namely young and male. It is obvious that in a complex social system assessing the impact of a WPP is difficult, as participants are always exposed to multiple information sources (media, family, etc.). We addressed this issue by including an open question asking participants what their main source of information regarding HIV and AIDS was in the survey. Most indicted electronic and printed media as their main source of information. More evidence is needed however, to determine how to construct WPPs to complement other campaigns to avoid cost-ineffective redundancy.

## Conclusions

We found good HIV-related knowledge among ministerial employees in Namibia. Differences between employees in the capital and in the regions were surprisingly low, with the exception of Caprivi. Several behavioral risk factors for HIV transmission persist in Namibian public employees such as multiple concurrent partnerships. They should be continuously targeted in national campaigns and educational programs as well as workplace programs with a special focus on remote high prevalence regions.
